# Rod–Coil Block Copolymer: Fullerene Blend Water-Processable Nanoparticles: How Molecular Structure Addresses Morphology and Efficiency in NP-OPVs

**DOI:** 10.3390/nano12010084

**Published:** 2021-12-29

**Authors:** Anna Maria Ferretti, Marianna Diterlizzi, William Porzio, Umberto Giovanella, Lucia Ganzer, Tersilla Virgili, Varun Vohra, Eduardo Arias, Ivana Moggio, Guido Scavia, Silvia Destri, Stefania Zappia

**Affiliations:** 1 Laboratorio di Nanotecnologie, Istituto di Scienze e Tecnologie Chimiche “Giulio Natta” (SCITEC)—CNR, Sezione Via G. Fantoli 16/15, 20138 Milano, Italy; 2 Istituto di Scienze e Tecnologie Chimiche “Giulio Natta” (SCITEC)—CNR, Sede Via A. Corti 12, 20133 Milano, Italy; marianna.diterlizzi@scitec.cnr.it (M.D.); william.porzio@scitec.cnr.it (W.P.); umberto.giovanella@scitec.cnr.it (U.G.); guido.scavia@scitec.cnr.it (G.S.); silvia.destri@scitec.cnr.it (S.D.); 3 Istituto di Fotonica e Nanotecnologie (IFN)—CNR, P.zza Leonardo da Vinci 32, 20132 Milano, Italy; lucia.ganzer@polimi.it (L.G.); tersilla.virgili@ifn.cnr.it (T.V.); 4 Department of Engineering Science, University of Electro-Communications, 1-5-1 Chofugaoka, Chofu, Tokyo 182-858, Japan; varun.vohra@uec.ac.jp; 5 Centro de Investigación en Química Aplicada (CIQA), Boulevard Enrique Reyna 140, Saltillo 25294, Mexico; eduardo.arias@ciqa.edu.mx (E.A.); ivana.moggio@ciqa.edu.mx (I.M.)

**Keywords:** PCPDTBT, miniemulsion, water-processable nanoparticles, OPV, nanodomain, TEM, EFTEM

## Abstract

The use of water-processable nanoparticles (WPNPs) is an emerging strategy for the processing of organic semiconducting materials into aqueous medium, dramatically reducing the use of chlorinated solvents and enabling the control of the nanomorphology in OPV active layers. We studied amphiphilic rod-coil block copolymers (BCPs) with a different chemical structure and length of the hydrophilic coil blocks. Using the BCPs blended with a fullerene acceptor material, we fabricated NP-OPV devices with a sustainable approach. The goal of this work is to clarify how the morphology of the nanodomains of the two active materials is addressed by the hydrophilic coil molecular structures, and in turn how the design of the materials affects the device performances. Exploiting a peculiar application of TEM, EFTEM microscopy on WPNPs, with the contribution of AFM and spectroscopic techniques, we correlate the coil structure with the device performances, demonstrating the pivotal influence of the chemical design over material properties. BCP5, bearing a coil block of five repeating units of 4-vinilpyridine (4VP), leads to working devices with efficiency comparable to the solution-processed ones for the multiple PCBM-rich cores morphology displayed by the blend WPNPs. Otherwise, BCP2 and BCP15, with 2 and 15 repeating units of 4VP, respectively, show a single large PCBM-rich core; the insertion of styrene units into the coil block of BCP100 is detrimental for the device efficiency, even if it produces an intermixed structure.

## 1. Introduction

In the last few decades, organic photovoltaics (OPVs) have attracted considerable interest as a promising alternative energy source, especially considering the capability of this technology to produce lightweight, flexible, and semitransparent devices using solution-processable coating and printing processing for large-scale production [1]. The efficiency issues of OPV compared to silicon devices have fallen due to the recent record results of single-junction OPV [2] in thick film-based [3] and semitransparent OPV [4], and in engineering from materials to devices, which paves the way for large area device development [5]. Nevertheless, OPV’s industrial implementation is still restricted by some relevant drawbacks [6,7].

In particular, OPV fabrication involves the use of large amounts of chlorinated organic solvents (e.g., chloroform, chlorobenzene, dichlorobenzene, etc.) on the laboratory scale in order to obtain effective active layer morphology with an optimized interpenetrating network between donor and acceptor materials [8]. The ideal industrial production should be highly sustainable, reducing the environmental impact and consequently the manufacturing cost of the devices [9,10].

Recently, the attention of different research groups has focused on the replacement of halogenated organic solvents in OPV device fabrication with more sustainable alternatives. However, it is very important to point out that despite being considered non-toxic, the most popular alternative solvents, such as toluene [11,12], xylene [13,14,15], tetrahydrofuran [16,17,18], and others [19,20,21,22], are not risk-free [23].

The production of water-processable nanoparticle (WPNP) dispersions of organic semiconducting materials emerged as an alternative approach for the fabrication of sustainable active layers. They reduce the use of chlorinated solvents, thus lowering the payback of the energy obtained from OPV devices, and at the same time they address the morphology of the device active layers [24]. The WPNP dispersions are obtained through the process into the alcoholic or aqueous medium of organic semiconductors that were designed to be soluble into organic solvents.

Two different methods have been reported in the literature. The nanoprecipitation approach allows one to obtain surfactant-free nanoparticles (NPs) in alcoholic medium with an efficiency of 4% [25]. Unfortunately, the alcoholic inks obtained show intrinsic instability [26]. The second one is the miniemulsion process, which involves the use of surfactants dissolved into aqueous phases that allow one to achieve stable inks [27].

The miniemulsion process, initially investigated by Landfester and co-workers [28,29], allows the formation of NPs of polymeric materials for preparing stable droplets of 50–500 nm, exploiting the shear forces applied on a system containing water, a highly hydrophobic compound dissolved into an organic solvent immiscible with water (chloroform, toluene, *o*-xylene, etc.) and the surfactant. This one protects the nanodroplets against collisions and mass exchanges, to obtain stable polymeric aqueous dispersions after the evaporation of the organic solvent. The increasing number of papers reporting on the application of this method in the production of OPV devices proves its versatility, involving a wide range of materials with performances of up to 3.8% [30] using sodium dodecyl sulfate (SDS) as the surfactant. The drawback of this method is that the SDS must be removed at the end of the preparation through dialysis. In 2018, the use of aqueous inks containing NP dispersions led to a record efficiency of 7.5% [31], exploiting a nanoprecipitation-assisted strategy with Pluronic F127 as the non-ionic surfactant. This method shows good potential for its wide application, and encouraged an in-depth study of nanoparticle OPV (NP-OPV) features in order to understand the basic principles behind this approach.

An additional advantage of the miniemulsion method is the fine control of the morphology of the NP-OPV active layer aided at each level of the device fabrication, without the addition of toxic solvent additives like 1, 8-diiodooctane or 1-chloronaphthalene. During the ink preparation, it is possible to control the domain segregation of the two active materials at the nanoscale level in order to achieve high charge carrier mobilities. Throughout the film deposition, the NP assembly enables one to build the active layer with an optimized morphology at the mesoscale, aiding effective exciton dissociation and free-charge transport and collection.

It is important to underline that the phase separation between the electron acceptor and donor materials leads to complex internal structures in the NPs. This is controlled by the inherent material properties, as long as the solvent evaporates. In particular, as reported for standard OPV devices, the surface energy plays a crucial role in the improvement of the miscibility of two blend components. Moreover, the surface energy affects the quality of the active layer’s morphology, enhancing the interfacial area between the materials with an efficient charge generation and dissociation, until the efficiency of the device is increased [32].

The benchmark electron acceptor material used in the WPNP dispersion fabrication is [6,6]-phenyl-C61-butyric acid methyl ester (PCBM), and this shows higher surface energy with respect to the semiconducting polymers. The high surface energy leads to core–shell NPs with fullerene derivatives in the core and electron donor polymers in the shell [33].

It is mandatory to consider other parameters for controlling the phase size and composition, which are related to material features and processing parameters, namely, the donor:acceptor ratio, the crystallinity and molecular weight of the donor material, the boiling point of the organic solvent in the miniemulsion process, and the post-deposition annealing treatment of the active layer [34].

Recently, we reported on the aqueous processing of four amphiphilic low band gap rod–coil block copolymers (BCPs), exploiting an adapted miniemulsion approach that avoids the use of surfactants for the preparation of neat water-processable nanoparticles (nWPNPs) [35]. A similar approach has been applied to the fabrication of NP-OPVs, adding PCBM to the production of the stable aqueous suspensions, achieving a peculiar NP inner morphology in the case of BCP5 bearing a coil block composed of five repeating units of 4-vynilpyridine (4VP), which produces working devices with a relevant photocurrent and efficiency up to 2.5% [36].

The use of amphiphilic BCPs for phase separation in the nanodroplets enriches the process with a further compartmentalization in the nanoparticles. BCPs display a well-known capability to self-assemble on the basis of the physical––chemical behavior of their components, to achieve phase separation at the nanoscale without segregation at the macroscopic level [37]. This feature attracts the interest of the researchers, suggesting a good candidate for electronic and optoelectronic applications. In particular, rod-coil BCPs constituted by a semiconducting polymer as the rigid block (for example, poly(3-alkylthiophene)) were tested in order to address the nanomorphology of OPV device active layers, in order to increase their efficiency [38,39,40,41].

In this study, the same method is applied to the preparation of blend water-processable nanoparticles (bWPNPs) composed of PCBM as the electron acceptor material, and different poly[2[2,6-(4,4-bis-(2-ethylhexyl)-4*H*-cyclopenta[2,1-*b*;3,4-*b*’]dithiophene)-*alt*-4,7(2,1,3-benzothiadiazole)] (PCPDTBT)-based rod–coil BCPs as the electron donor material, characterized by a standardized rod block, PCPDTBT, and tailored poly-4-vinylpyridine (P4VP)-based flexible segments with various lengths and molecular compositions. The coil blocks were prepared with 2 or 15 repeating units of 4-vinylpyridine (4VP), in the case of BCP2 and BCP15, respectively [42,43]; while for BCP100, a long coil block of a random copolymer poly(styrene-*co*-4-vinylpyridine) with 76% styrene and 24% of 4VP was obtained [42]. The ratio for the blend of PCBM:BCP in the starting organic solution was the same in each case, 3:1, as reported for OPV devices with similar active layers. The obtained results were compared with those obtained in the case of BCP5 [36].

We took advantage of the BCP’s ability to assemble into organized nanostructures suspended into aqueous medium [35], and we exploited the interaction of the P4VP-based flexible segments with the non-solvent aqueous phase to achieve stable blend water-borne nanoparticles in suspensions. Moreover, the effect of the coil length on the blend’s aggregation into the bWPNPs was evaluated, and the effect of the morphological features of bWPNP nanodomains on their performances in the devices was elucidated. In particular, we studied the bWPNP morphology by means of atomic force microscopy (AFM), transmission electron microscopy (TEM), and energy filtered TEM (EFTEM), to clarify how the morphology of the nanodomains is related to the features of the different coil molecular structures in the BCPs, and how they led to different device performances. An ultrafast spectroscopy study elucidated the charge separation dynamics in the film obtained from bWPNPs [44].

## 2. Materials and Methods

### 2.1. Materials

**Figure 1:**
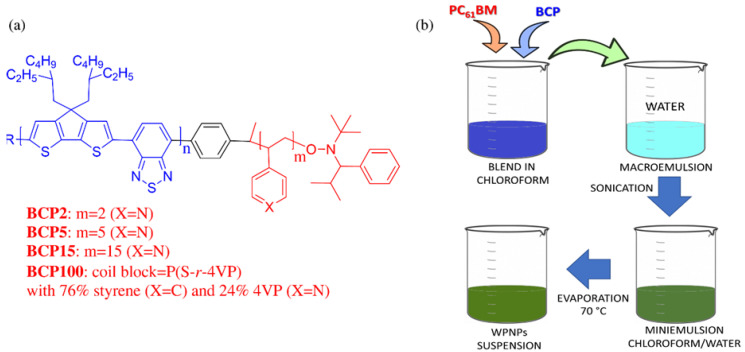
(**a**) Molecular structures of the amphiphilic rod–coil block copolymers (BCPs) used in this work; (**b**) preparation of the blended water-processable nanoparticles (bWPNPs) through the adapted miniemulsion approach as used in this work.

MilliQ water of ultrapure grade was used (resistivity of ~18 MΩ∙cm^−1^ at 25 °C) for the preparation of the water-processable nanoparticles (WPNPs). The molecular structures of the four amphiphilic rod–coil BCPs are depicted in Figure 1. The synthetic routes for BCP2, BCP5, BCP15 and BCP100 are reported in Scheme S1, and they have been assessed as already reported [35,45]. PCBM was purchased by Ossila and used without further treatment. All solvents were purchased from Sigma-Aldrich and used as received for the WPNP preparation. PEDOT:PSS PVP Al 4083 from Heraeus was used for the device fabrication.

### 2.2. Methods

#### 2.2.1. Preparation of PCBM:BCP of the Blend WPNPs

The blend WPNPs (bWPNPs) were prepared through the miniemulsion approach, using a modified Landfester procedure [29,46]. In a typical experiment, the two components of the blend were dissolved into chloroform in order to achieve two starting solutions with standard concentrations, 25 mg∙mL^−1^ and 10 mg∙mL^−1^, for PCBM and BCP, respectively. A proper quantity of each starting organic solution was mixed up to obtain an organic solution of the blend with an actual ratio of PCBM:BCP = 3:1. Then, 200 µL of the blend organic solution was sonicated, warmed at 40 °C, and slowly poured into 1 mL of pre-heated MilliQ water at 40 °C, under vigorous stirring. After ~60 min the macroemulsion was sonicated for 10 min at 40 °C in an ultrasonic bath to achieve stable miniemulsion that was heated up to 70 °C and gently stirred to completely remove the organic solvent and to get brown-greenish stable aqueous suspensions of bWPNPs. All steps of the preparation were performed in air.

#### 2.2.2. Optical and Electrical Characterization of the PCBM:BCP = 3:1 Blend WPNPs

UV–visible absorption spectra were recorded with a Perkin Elmer Lambda 900 spectrometer (Perkin Elmer, Waltham, Massachusetts). Optical characterizations were performed on aqueous suspensions and films. The aqueous samples were drop-casted on plasma-treated glass substrates, then dried at 60 °C for 30 min. The annealed samples were treated at 90 °C under nitrogen flux for 20 min. The cooling process was performed under a nitrogen atmosphere.

The hydrodynamic diameter and ζ potential of bWPNPs were determined through dynamic light scattering (DLS) using a Brookhaven 90 Plus size analyzer (Brookhaven Instruments Corporation, Holtsville, NY, USA). The apparatus was equipped with a He–Ne laser emitting light at λ = 632.8 nm and a detector recording intensity at a fixed scattering angle θ = 90°. All measurements were performed at room temperature. Samples for the measurements were prepared by diluting the original WPNP suspensions 1/30 with MilliQ water.

Mobility Measurements. The hole-only devices with an ITO/active layer/Ag architecture were fabricated to guarantees unipolar hole charge injection into the active layer. The space–charge limited current (SCLC) method was modeled with the Mott–Gurney equation and [47].

Pump–probe experiments were performed with a fraction of the output light of a Ti:sapphire laser (Libra, Coherent) characterized by 100 fs pulse duration, 1 kHz repetition rate and 800 nm central wavelength. Pump excitation wavelengths of 600 nm were used for the characterization of the blend sample. The 600 nm light came from a non-collinear optical parametric amplifier. The broadband probe extending in the visible region resulted from white light continuum generation in a 3 mm-thick sapphire plate pumped with 800 nm light. The delay between pump and probe pulses was controlled by a translation stage and the pump beam was modulated by a mechanical chopper with a 500 Hz frequency. The differential transmission (ΔT/T) of the probe was measured as a function of probe wavelength and pump–probe delay through an SP2150 Acton spectrometer from Princeton Instruments The pump energy was adjusted to a fluence around 45 μJ cm^−2^. Measurements were performed with parallel polarizations between pump and probe beams.

#### 2.2.3. Fabrication and Characterization of the OPV Devices

Patterned indium tin oxide (ITO) substrates were purchased from Atsugi Micro. The ITO substrates were cleaned using a standard procedure by sequential ultrasonication in acetone, detergent, ultrapure water, isopropanol and hot isopropanol. After exposure to ultraviolet ozone (UV/O_3_) treatment for 5 min, the substrates were coated with a poly(3,4-ethylenedioxythiophene)-polystyrene sulfonate (PEDOT:PSS) by spin-coating an aqueous dispersion (Heraeus Clevios PVP AI 4083) at 5000 rpm for 30 s, followed by annealing at 150 °C for 20 min, and the PCBM:BCP WPNP suspension was spin-coated onto UV/O_3_-treated ITO/PEDOT:PSS substrates at 500 rpm for 90 s. The samples were then dried at 60 °C for 30 min in air prior to spin-coating a PCBM solution (10 mg·mL^−1^ in dichloromethane) at 4000 rpm for 10 s on top of the multilayer. After drying the active layer at 90 °C for 20 min, the substrates were placed in a high vacuum overnight prior to the sequential thermal evaporation of bathocuproine (10 nm) and Ag (65 nm) to finalize the devices. The device area, defined by the cross-section of the ITO and Ag electrodes, was of 0.02 cm^2^. The J-V characteristics were measured under simulated sunlight (AM1.5G, 100 mW·cm^−2^) using a Keithley 2401 sourcemeter. The photovoltaic parameters presented in this study correspond to the average of 4 devices and exhibited a minor deviation below 5%.

#### 2.2.4. Morphological Characterization of the PCBM:BCP = 3:1 Blend WPNPs

Grazing incidence wide-angle X-ray scattering (GIWAXS) measurements were performed at the X-ray Diffraction beamline 5.2 at the Synchrotron Radiation Facility Elettra in Trieste (Italy). The X-ray beam emitted by the wiggler source on the Elettra 2 GeV electron storage ring was monochromatized by a Si (111) double crystal monochromator, focused on the sample and collimated by a double set of slits giving a spot size of 0.2 × 0.2 mm^2^ (λ = 0.14 nm). The samples were oriented by means of a four-circle diffractometer with a motorized goniometric head. The X-ray beam direction was 3 fixed, while the sample holder could be rotated about the different diffractometer axes, to reach the sample surface alignment in the horizontal plane containing the X-ray beam. Film opacity prevented laser-assisted sample alignment, which was carried out with optical devices (camera). Bidimensional diffraction patterns were recorded with a 2M Pilatus silicon pixel X-ray detector (DECTRIS Ltd., Baden, Switzerland) positioned perpendicular to the incident beam, at a variable distance (180 up to 200 mm distance from the sample), to record the diffraction patterns in the reflection mode. The sample and detector were kept fixed during the measurements. The q range of diffracted patterns was estimated by means of Lanthanum hexaboride powders (standard reference material 660a of NIST) and it was evaluated ranging up to 0.27 or 0.38 nm^−1^, depending on sample to detector distance, in agreement with similar synchrotron GIWAXS measurements. Measurements were performed under He-flux in a properly adapted glove box to prevent both damaging and air scattering contribution to background. Data were processed using GIDVis software.

Atomic Force Microscopy (AFM, NT-MDT Spectrum Instruments, Moscow, Russia) was performed with commercial equipment (AFM NTMDT NTEGRA) in tapping mode with a cantilever NSG10 operating at a typical resonance frequency of 140–390 kHz. The samples were prepared using glass slides properly cleaned with plasma treatment for 10 min before the deposition of the aqueous bWPNP suspensions. The as-prepared samples were dried at 60 °C in air and then annealed at 90 °C for 20 min in nitrogen flux.

Transmission Electron Microscopy (TEM) and EFTEM images were collected by means of a 200 kV ZEISS LIBRA 200 FE microscope equipped(Carl Zeiss Microscopy, Oberkochen, Germany) with a second generation column Ω filter, and the EFTEM images were recorded by centering the energy selecting slit at 22 eV and 30 eV with a ±2 eV range. The four samples were prepared following this procedure: the mother solution was diluted 1:3 with MilliQ water then a 7 µL drop of bWPNP suspension was dropped on a copper grid covered by a SiO film pre-treated with a plasma cleaner; the excess of water was blotted with filter paper after 1 min. The estimation of the WPNP diameter measurement was performed using TEM Imaging Platform Olympus and dm = ∑d_i_n_i_/∑n_i_, where ∑n_i_ is the number of particles of dimension d_i_ [48].

## 3. Results and Discussion

### 3.1. Design of the Materials and Blend Water-Processable Nanoparticle (bWPNP) Suspension Preparation

Four amphiphilic rod–coil BCPs were studied in this work, whose molecular structures are shown in Figure 1a. They were composed of a standardized low-band gap (LBG) copolymer (poly[2,6-(4,4-bis-(2-ethylhexyl)-4*H*-cyclopenta[2,1-*b*;3,4-*b*’]dithiophene)-*alt*-4,7-(2,1,3-benzothiadiazole)]) (PCPDTBT) as rod block, synthetized with a Suzuki polycondensation with 10–12 dimeric units [35,43]. The flexible segments were tailored P4VP-based polymers, namely, each BCP had 2, 5 or 15 repeating units of 4-vinylpyridine (4VP) for BCP2, BCP5 or BCP15, respectively [35]. A segmented random copolymer of styrene and 4VP (with a composition of 76% of styrene and 24% of 4VP) P(S-*r*-4VP) for BCP100 was in turn synthetized with a nitroxy-mediated radical polymerization [35,45]. The hydrophobic behavior of the rod block PCPDTBT and the capability of the P4VP-based coil blocks to interact with the aqueous medium provided amphiphilic abilities to the BCPs, stabilizing the aqueous/non-aqueous interfaces during WPNP fabrication. BCPs can interact with fullerene derivatives, the electron acceptor material used in OPV active layer, by means of the lone pairs on the nitrogen on the pyridine rings [45]. In the case of BCP100, styrene units were inserted in the coil with the aim of increasing the quality of the deposition of the aqueous inks.

Considering the BCP features, our approach to formulate water-processable nanoparticles (WPNPs) was based on the miniemulsion technique without the use of surfactants, and consequently no additional purification steps were required to remove the excess of surfactant used, as sketched in Figure 1b. Accordingly, this approach simplified and reduced the cost of the WPNP fabrication with respect to the surfactant-based miniemulsion process. Noticeably, the morphology and stability of the obtained suspensions were determined by the BCP molecular structures, and particularly by the coil one. Moreover, this technique ensures almost stable WPNP dispersions are achieved, with a core–shell organization that could be retained during the film’s deposition on whatever substrate.

To achieve nanoparticle suspension for the active layer deposition, we prepared the blend of PCBM:BCP with a controlled ratio of 3:1 solubilized into chloroform. This is a good, low-boiling point solvent for both the blocks. The concentration of the organic starting solution was maintained in each formulation for both blend components to compare the different samples in a homogeneous way. The organic solution was processed as described in Section 2.2.1 and shown in Figure 1b. At the end of the process, stable greenish/brownish aqueous suspensions were achieved and used without further purification.

### 3.2. Characterization of the Blend WPNP Suspensions

**Figure 2:**
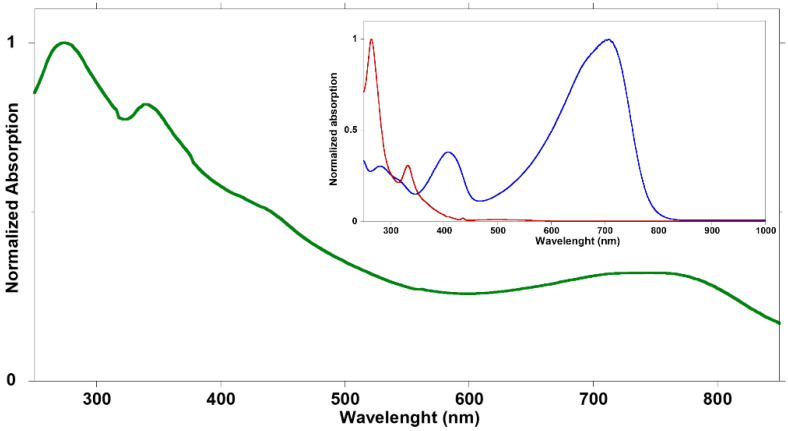
Normalized absorption spectrum of BCP15 bWPNPs dispersion after dilution with water 1:100 (green line); in the inset normalized spectra of BCP15 (blue line) and PCBM (red line) dissolved in chloroform.

The aqueous suspensions were optically characterized through UV–vis absorption spectroscopy. Figure 2 shows the absorption spectrum of BCP15 bWPNP dispersion. The spectra of the other samples are reported in the supplementary materials (Figure S1). The spectrum shows the typical bands attributable to the two components of the blend (Figure 2, inset), covering the entire visible spectrum, from 250 to 850 nm. Two peaks at about 410 and 700 nm due to the rod block are detected. The broadening of the peaks is due to the aggregation of the polymeric backbones into the suspended nanostructure. Moreover, the addition of the fullerene derivatives to the blend leads to the further broadening and red-shifting of the absorption peaks.

To derive information about the size and stability of the bWPNPs suspended in aqueous medium, dynamic light scattering (DLS) was carried out. Unfortunately, the determination of the hydrodynamic diameter (d_H_) was quite complex due to the wide size distribution of the suspended bWPNPs. In Table 1, the size distribution by number of each sample is reported and compared with data obtained from samples prepared with BCP neat WPNPs (nWPNPs), i.e., without the insertion of PCBM [35], considering that the distribution by number is the closest to the analysis of the size obtained by transmission electron microscopy (TEM).

### 3.3. Blend WPNP-Based OPV Device Characterization

As in the case of BCP5 [36], the vertical charge carrier mobility was preliminarily measured in hole-only devices with an ITO/neat BCP-based NPs/Ag architecture through the SCLC method. The hole mobility was investigated through the SCLC method (µ^h^_SCLC_) in films fabricated with BCP nWPNPs, prepared with a thickness of around 100–150 nm, before and after thermal treatment for 20 min (Table S1).

The µ^h^_SCL_ of BCP15 nWPNPs increased one order of magnitude in the treated samples comparing to the pristine one (from 1.5 × 10^−4^ to 3 × 10^−3^ cm^2^V^−1^s^−1^, respectively), due to the improved interconnections upon annealing among nanostructures, so that the percolative pathway formation for the collection of the charges was promoted. A lower µ^h^_SCL_ was observed for BCP100 nWPNPs, even if the quality of the films was higher. The insertion of a long flexible coil in the BCP100 backbone composed of a high percentage of styrene units hampered the collection of the charges in the films. In the case of BCP2 nWPNPs, a resistive behavior was observed both before and after thermal treatments, up to 120 °C. This behavior was attributed to the non-continuous film, confirmed by the topological analysis (see below), formed by the direct deposition of the suspension.

**Figure 3:**
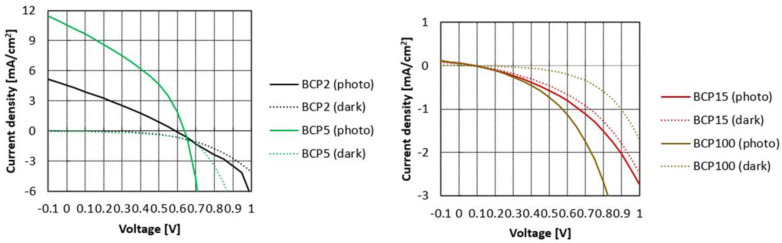
J−V curves of the devices based on BCP2 and BCP5 bWPNP dispersions (**left**) and on BCP15 and BCP100 bWPNP dispersions (**right**).

Considering the relevant efficiency obtained in the case of BCP5 bWPNPs, we fabricated OPV devices in a direct configuration using the aqueous inks for the preparation of the active layers and after suitable pre-deposition treatments [36,49]. A thin buffer layer of PCBM deposited from dichloromethane to seal the active layer was dropped before the thermal treatment of the devices. For each blend suspension, four devices were fabricated and characterized. These results correspond to an average of the performances of these devices. All devices showed relatively small variations with an error of less than 5%. Typical current density–voltage (J–V) curves fabricated with the BCP2, BCP5, BCP15 and BCP100 bWPNP dispersions are displayed in Figure 3, and the average device performances for each sample are reported in Table 2.

The figures of merit of the devices were lower than those obtained in the case of the BCP5 bWPNP devices, which showed a PCE of 2.49%, with a high photocurrent of 10.61 mA/cm^2^. In the case of the BCP2 bWPNPs, an efficiency lower than 1% was obtained with a photocurrent of around 4.5 mA/cm^2^, whilst in the case of the BCP15 and BCP100 bWPNPs, the PCE decreased two orders of magnitude, as did the other parameters.

Considering the chemical composition of the rod–coil block copolymers, it is possible to investigate how the length and the structure of the coil block influences the features of the fabricated devices, leading to a dramatic drop in the efficiency.

### 3.4. The Characterization of the PCBM:BCP = 3:1 bWPNP Film

The crystallization tendency of PCPDTBT bearing branched alkyl side groups, as in the rod block of the BCPs of this work, is negligible, in particular without the use of high-boiling point solvent additives [50]. To investigate the macromolecular organization of the bWPNPs in the active layer, GIWAXS measurements of treated films were performed and are reported in detail in the supplementary materials. The initial low degree of order obtained from sample preparation was not substantially improved by the same thermal annealing as that adopted in the device preparation; it was necessary to repeat the annealing to achieve some ordered packing. The main features are the inter-macromolecular spacing near 0.9 nm, while the main peak attributable to PCBM completely overlaps both the film substrate and the bump of the amorphous part. At the molecular level, it is clear that crystal packing differences among various copolymers are almost negligible in the bWPNP films too.

**Figure 4:**
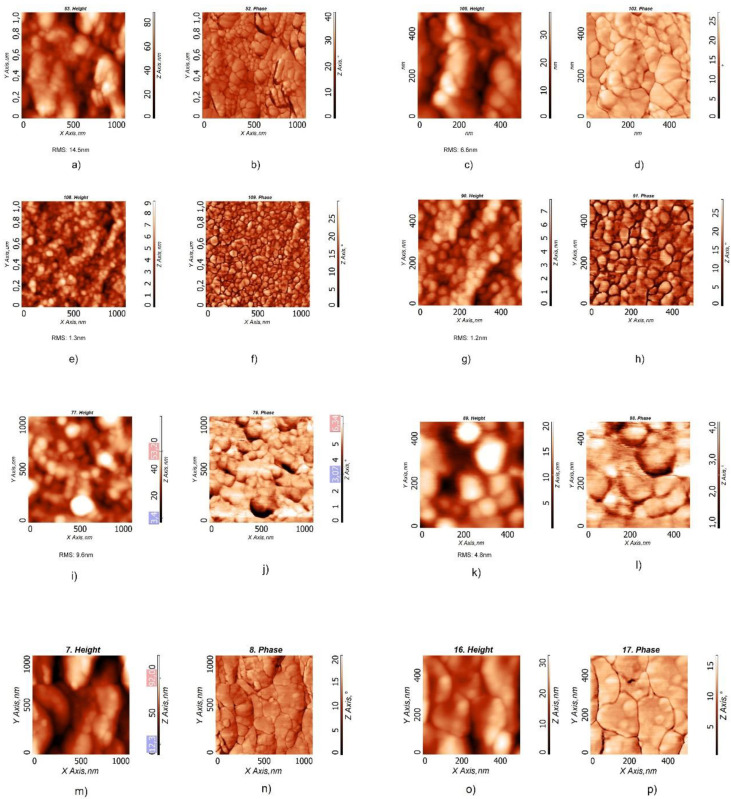
AFM images of films obtained from the aqueous dispersions, with two different magnifications, of BCP2 bWPNPs (**a**–**d**), BCP5 bWPNPs (**e**–**h**), BCP15 bWPNPs (**i**–**l**) and BCP100 WPNPs (**m**–**p**).

The films, annealed at 90 °C for 20 min, were investigated with atomic force microscopy (AFM) (Figure 4). All the samples showed a distribution of spherical particles, highly clustered, but the obtained layers were different as a function of the sample used. The layers obtained from the BCP2 and BCP100 bWPNPs dispersions (Figure 4a–d and Figure 4m–p, respectively) were discontinuous, while in the case of the BCP5 bWPNPs (Figure 4e–h) and, to a lesser extent, in the case of the BCP15 bWPNPs (Figure 4i–l), the NPs led to well-assembled and compact layers. The root mean square (RMS) values reveal that the layers obtained by the BCP5 bWPNP dispersions have the minimum roughness value, compared with those measured for the BCP2, BCP15 and BCP100 bWPNP dispersions (Table 3), confirming the higher quality of the BCP5 bWPNP film in terms of homogeneity and compactness (Figure 4e–h).

### 3.5. Morphological Investigation of bWPNP Suspended in Aqueous Inks

**Figure 5:**
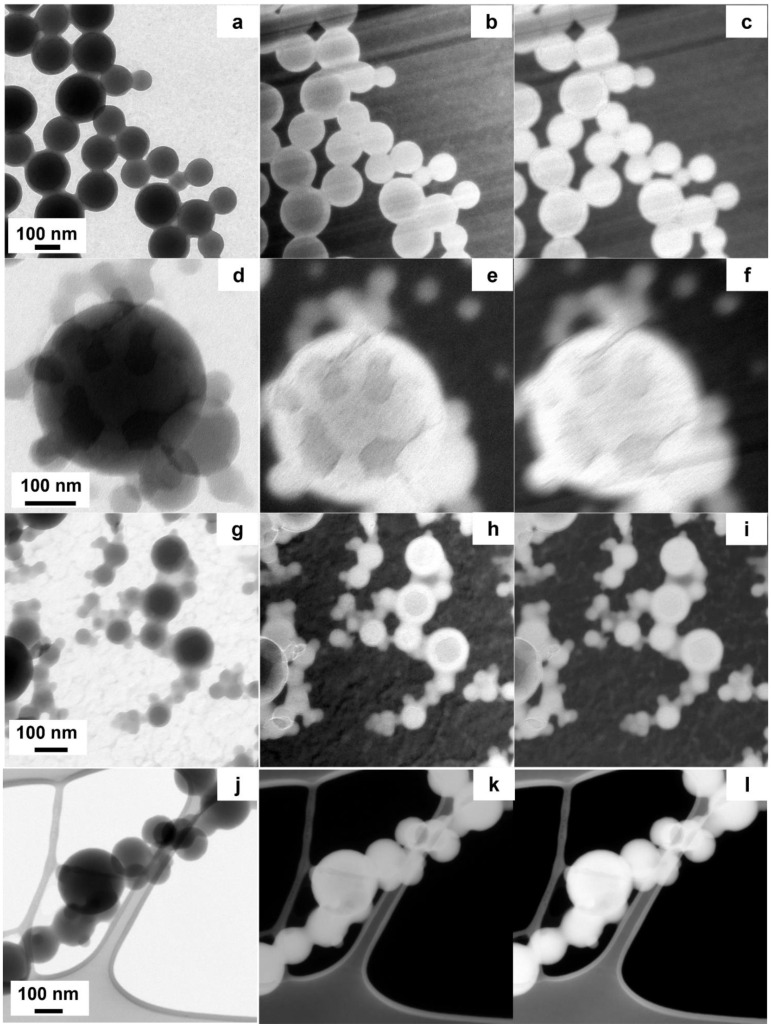
TEM and EFTEM images of bWPNPs are reported: (**a**,**d**,**g**,**j**) show the conventional TEM image, (**b**,**e**,**h**,**k**) are the EFTEM images recorded at 20 eV, (**c**,**f**,**i**,**l**) are the EFTEM images collected at 30 eV.

We investigated the morphology of the WPNPs at the nanoscale level, to demonstrate if there is a relationship between the material chemical structure (the coil structure) and the formation of suitable donor/acceptor domains inside the bWPNPs, as observed in the case of BCP5 bWPNPs. The TEM micrographs showed that the studied bWPNP samples have a spherical shape (Figure 5a,d,g,l). The mean diameters of the bWPNP samples, measured from TEM micrographs, are larger than the corresponding nWPNP diameters [31]. The mean diameters are reported in Table 4 and their size distribution is reported in Figure S3. The WPNP size increase is clearly related to the presence of the PCBM blended with the BCP. The absence of precipitate observed during the miniemulsion process supports the hypothesis that the interactions between the electron donor and acceptor organic components are effective. Moreover, it is known that the PCBM has a higher surface energy than the rod block PCPDTBT (45.8 and 40.5 mN/m, respectively) [51], and we expect that PCBM will tend to fold in the bWPNP core [33,34], contributing to increasing the NP size, as previously observed. Based on these remarks, it is reasonable to hypothesize the presence of PCBM-enriched domains in the bWPNP core. In addition to the morphology characterization, the identification of the shape and distribution of the nanodomains inside bWPNPs is essential to understanding the nanostructure behavior. The TEM has the correct length–scale resolution, but it has a very low contrast in the case of materials with very low and similar atomic numbers. Fortunately, semiconducting polymers are usually rich in covalent bonds, so different organic components can be identified by means of EELS. In particular, the low-loss region of these EELS spectra gives important information about the dielectric properties of the studied nanostructures. To correlate this information with the spatial resolution, we exploited the EFTEM imaging. The EFTEM imaging increases the image contrast, exploiting the intrinsic difference in electronic structure between BCP and PCBM [52]. The EELS spectra showed that the plasmon peak of BCP has a maximum around 22 eV, similar to most carbon-based polymers [53], while it is known from the literature that the PCBM plasmon peak has a maximum at 30 eV [36,54]. In order to identify the size and distribution of the PCBM nanodomains inside the bWPNPs, we recorded the EFTEM images at 22 eV and 30 eV. To select the correct energy to filter the image, we first recorded the low-loss EELS spectrum of the nWPNP and bWPNP samples. We noticed that the bWPNP plasmons (~25–26 eV) shifted to greater losses of energy with respect to the nWPNP ones (Figure S4), but these were lower with respect to the pure PCBM plasmon. This energy loss lowering was caused by the superimposition of the BCP plasmon signal with the PCBM one. To discriminate the two signals’ contributions to EFTEM images, we recorded them at 22 eV (Figure 5b,e,h,k) and 30 eV (Figure 5c,f,i,l). In the micrographs collected at 22 eV, the BCP-rich areas are lighter, while the PCBM-enriched ones are darker. On the contrary, the latter becomes lighter once collected at 30 eV, while the BPC brightness does not decrease significantly, because of the BCP plasmon contribution to the 30 eV EFTEM image. As shown in Figure 5b,h, the BCP2 and BCP15 bWPNPs show similar single and large-size cores enriched in PCBM, at 120.6 ± 26.1 nm and 76.5 ± 47.4 nm, respectively, and BCP-based shells. The core size distributions are reported in Figure S5. The internal structure of the WPNPs can be described as a three-level structure: a single internal core enriched in PCBM, and in the other hydrophobic components of the BCP, a second level around the core enriched in BCP, and a third thin shell where the hydrophilic coils substitute the surfactant stabilizing the WPNPs in an aqueous medium [35]. This model justifies the apparently unexpected larger size of the BCP2 bWPNPs. The short coil of BCP2 is not as good a stabilizer as the longer coils of the BCP5 and BCP15 samples. Consequently, during the emulsion process of the BPC2 blend, the drops coalesce to form larger hydrophobic drops. They reduce the surface area, given that in the BCP2, the coil is short and has lower stabilizing power. The described internal morphology, with large and regular cores, agrees with the low device efficiency of these two samples; actually, the single domain core is too large to be efficient, and the BCP shell is too thick to allow domain connection, even after thermal treatment.

The other two samples displayed a different domain morphology. BCP100 bWPNP does not show any fullerene domains, because the PCBM is uniformly distributed inside the bWPNP (Figure 5l–n). The BCP100 molecular structure, with the presence of a high percentage of hydrophobic styrene in the coil block, improves the homogeneity in the nWPNPs [35]. In the BCP100 bWPNPs, the fullerene units interact in the same way with the rod as with the coil because of the higher hydrophobicity of the coil block with respect to the other samples. Hence, the uniform distribution of the PCBM inside the BCP100 WPNPs was detected, and no domains were identified. The intermixing of donor and acceptor materials is too thick to enable a good efficiency of the devices, and the low device performance is correlated with the photophysics of the bWPNP active layers. On the contrary, the BCP5 bWPNPs show many small PCBM-rich domains inside each bWPNP (Figure 5j–l). This internal morphology, with nanodomain size comparable to the light wavelength, contributes to the efficiency of the BCP5 bWPNP-based device [36].

### 3.6. Photophysical Investigation of bWPNP-Based Films

The pump–probe measurements were performed to study the photophysics of the BPC bWPNPs and to gain information on the electron donor–acceptor material charge transfer.

The pump–probe spectra of the spin-coated BCP2, BCP15, and BCP100 bWPNPs, respectively, have been previously reported [44]. A clear correlation has been highlighted between the formation of free charge generation at donor/acceptor interfaces and the length and nature of the coil blocks. In particular, in all samples, the photogenerated excitons in bWPNPs migrate in tens of picoseconds to the donor/acceptor interface to be separated into free charges. The more the coil length increases, the more long-living charge transfer states are formed, which leads to a non-efficient exciton separation mechanism.

**Figure 6:**
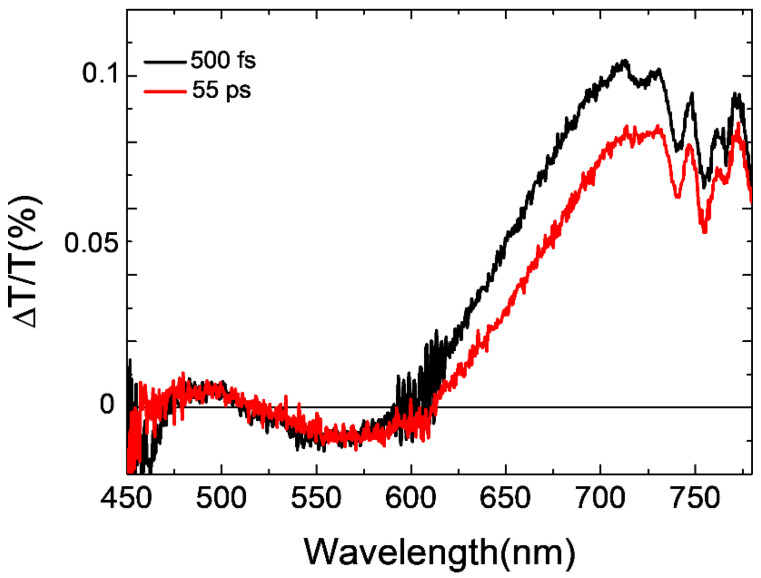
Pump and probe spectra at different probe delays for the BPC5 bWPNPs.

In order to confirm these remarks, Figure 6 shows the pump probe spectra of the spin-coated BCP5 bWPNP sample annealed at 90 °C for 20 min at 0.5 and 55 ps probe delays. As reported in [44] for the other blends, the spectra are dominated by a photoinduced absorption band at 550 nm and two positive bands at around 680 nm and 470 nm. The photoinduced spectral bands have been attributed to the PCPDTBT excitons: a photoinduced absorption band related to singlet–singlet absorption and two positive ground state bleaching (GSB) bands. We see the formation of a photoinduced absorption band at around 650 nm assigned to the electron transfer from PCPDTBT moieties to PCBM [44,55]. This is clear evidence that the short coil length (five repeating units) allows for an efficient charge transfer in the donor/acceptor interface without the deleterious formation of the charge transfer state [44]. The photophysical characterization confirmed a similar charge transfer efficiency between the BPC2 bWPNPs and the BPC5 bWPNPs. The different figures of merit for the device performance are more likely due to the presence of defects in/traps for charges along the films.

## 4. Conclusions

In summary, we synthetized four amphiphilic rod–coil block copolymers (BCPs), characterized by a standard rigid block constituted by a low band gap polymer, PCPDTBT, and tailored flexible blocks differing in length and chemical composition. We used BCP2, BCP5 and BCP15, bearing short segments of 4VP repeating units (2, 5, and 15, respectively), and BCP100, constituted by a long coil block of 76% styrene and 24% 4VP. The BCPs were used for the preparation of blend water-processable nanoparticles (bWPNPs) with PCBM as the acceptor material through an adapted miniemulsion approach, without using any other surfactants. The aqueous inks obtained were stable and were exploited for the fabrication of sustainable nanoparticle-based organic photovoltaics (NP-OPV). The device efficiency of the different materials was deeply influenced by the coil molecular design. We investigated the morphology of the water-borne nanoparticles by means of TEM, EFTEM and EELS to shed a light on the internal morphology of the nanostructures and correlate the dimensions of the donor/acceptor nanodomains with the performances of the devices. We investigated the photophysics of the bWPNP deposited on thin films in order to elucidate the charge generation process in the bWPNPs active layers.

BCP5 bWPNPs aqueous dispersions enabled us to gain working devices with similar efficiencies with respect to the devices obtained through organic solution processing, due to a relatively short coil block that allows the formation of many small PCBM-rich domains inside each bWPNP. Moreover, the efficient exciton separation mechanism at the donor/acceptor interfaces permitted a good charge generation that efficiently reached the electrodes.

A short coil block, as in the case of BCP2 bWPNPs, produced a core–shell morphology with PCBM-rich large cores. Even if the exciton separation was efficient, the low quality of the obtained films led to low device efficiency.

On the other hand, longer coil segments, as in the cases of BCP15 and BCP100, generated longer-lived charge transfer states that, together with the inefficient morphology of the nanodomains in the bWPNPs, justified the poor efficiencies achieved. In the case of BCP100, the presence of styrene in the coils with its insulating behavior was detrimental to the device’s efficiency.

In concluding, a careful molecular design is essential in the optimization of BCP molecular structures to enable morphology control in NP-OPV active layer fabrication and the optimization of efficient sustainable and eco-compatible OPV devices.

## Figures and Tables

**Table 1:** Hydrodynamic diameters (d_H_) expressed in the number and ζ potential of the nWPNP and bWPNP suspensions.

Sample	Hydrodynamic Diameter (d_H_) (nm)	ζ Potential (mV)
BCP2 nWPNPs	64.6 ± 1.5	−31.33 ± 0.37
BCP5 nWPNPs	90.2 ± 22.7	−19.05 ± 1.47
BCP15 nWPNPs	110.7 ± 1.2	−31.23 ± 1.14
BCP100 nWPNPs	92.3 ± 1.2	−26.58 ± 0.55
BCP2 bWPNPs	187.0 ± 2.5	−45.90 ± 0.60
BCP5 bWPNPs	133.8 ± 9.2	−14.75 ± 4.11
BCP15 bWPNPs	146.1 ± 2.2	−38.40 ± 1.14
BCP100 bWPNPs	129.6 ± 1.7	−34.33 ± 0.42

**Table 2:** Figures of merit of the devices obtained with PCBM:BCP = 3:1 bWPNP active layers.

Sample	Jsc (mA∙cm^−2^)	Voc (mV)	FF (%)	PCE ^a^ (%)
BCP2 bWPNPs	4.56	593	28.5	0.77 ± 0.03
BCP5 bWPNPs	10.61	638	36.7	2.49 ± 0.05
BCP15 bWPNPs	0.056	95	17.0	0.0009 ± 0.00
BCP100 bWPNPs	0.053	79	25.9	0.0012 ± 0.00

^a^ Average of 4 OPV devices.

**Table 3:** Root mean square (RMS) values corresponding to the BCP2, BCP5, BCP15, and BCP100 bWPNPs (Figure 4a,e,i,p).

Sample	Root Mean Square (RMS) (nm)
BCP2 bWPNPs	14.5
BCP5 bWPNPs	1.3
BCP15 bWPNPs	9.6
BCP100 bWPNPs	17.0

**Table 4:** Comparison of WPNP size, measured through TEM.

Sample	Mean Diameter (dm) (nm)	Std Dev (nm)	Min Diameter (nm)	Max Diameter (nm)
BCP2 nWPNPs	35.9	21.3	11.9	144.7
BCP2 bWPNPs	149.4	44.8	74.0	368.1
BCP5 nWPNPs	51.5	36.7	16.2	287.9
BCP5 bWPNPs	58.2	21.4	23.3	166.0
BCP15 nWPNPs	68.8	31.9	6.2	202.9
BCP15 bWPNPs	99.2	48.7	31	275
BCP100 nWPNPs	48.7 87.1	23.7 30.0	13.6	158.4
BCP100 bWPNPs	202.2	81.7	75.0	328.2

## Data Availability

Not applicable.

## Supplementary Materials

The following are available online at https://www.mdpi.com/article/10.3390/nano12010084/s1, Scheme S1. Synthetic routes for the BCPs used in this work (8 BCP2, 9 BCP5, 10 BCP15, and 11 BCP100, respectively); Figure S1. Normalized absorption spectra of BCP2 (green), BCP 5 (red) and BCP100 (blue) bWPNPs dispersion after dilution with water 1:100; Figure S2. XRD profile extracted from 2D images of BCP15 bWPNP film with 5 layers, vertical bars indicate diffraction features; Figure S3. Size distribution of the bWPNPs: (a) BCP2 bWPNPs; (b) BCP5 bWPNPs; (c) BCP15 bWPNPs; (d) BCP100 bWPNPs; Figure S4. EELS low-loss spectra. EELS spectra of the nWPNP sample (red), the plasmon peak is at 22 eV. EELS spectra of a BWPNP sample (black), where the plasmon peak is shifted to 25–26 eV because of the superimposition of the nWPNP sample and the PCBM plasmon that has its maximum at 30 eV. Figure S5. Size distribution of the PCBM-rich cores measured for the (a) BCP2 bWPNPs and (b) BCP15 bWPNPs; Table S1. Features of BCP blend WPNP films differently treated; Table S2. Hole mobility calculated through space–charge-limited current method of layers deposited from BCP nWPNP suspensions.
