# Supplementary material for: Rod–Coil Block Copolymer: Fullerene Blend Water-Processable Nanoparticles: How Molecular Structure Addresses Morphology and Efficiency in NP-OPVs
Source: Nanomaterials (Basel). 2021 Dec 29;12(1):84. doi: 10.3390/nano12010084 (PMC8746663; doi:10.3390/nano12010084)
Supplement: Supplementary file 1 [file nanomaterials-12-00084-s001.zip › nanomaterials-1508452-supplementary.pdf]

Supplementary Materials for

Rod-coil block copolymer:fullerene blend water-processable nanoparticles: how molecular structure addresses morphology and efficiency in NP-OPVs

Anna Maria Ferretti,[1*] Marianna Diterlizzi,[2] William Porzio,[2] Umberto Giovanella,[2] Lucia Ganzer,[3] Tersilla Virgili,[3] Varun Vohra,[4] Eduardo Arias,[5] Ivana Moggio,[5] Guido Scavia,[2] Silvia Destri[2] and Stefania Zappia.[2*]

[1] Istituto di Scienze e Tecnologie Chimiche "Giulio Natta" (SCITEC) - CNR, Laboratorio di nanotecnologie, via G. Fantoli 16/15, 20138 Milano, Italy

[2] Istituto di Scienze e Tecnologie Chimiche "Giulio Natta" (SCITEC) - CNR, via A. Corti 12, 20133, Milano, Italy

[3] Istituto di Fotonica e Nanotecnologie (IFN) - CNR, P.zza Leonardo da Vinci 32, 20132 Milano, Italy

[4] University of Electro-Communications, 1-5-1 Chofugaoka, Chofu, Tokyo 182-858, Japan

[5] Centro de Investigación en Química Aplicada (CIQA), Boulevard Enrique Reyna 140, 25294 Saltillo, México

The four amphiphilic block copolymers studied in this work were synthetized by the procedure depicted in **Scheme S1**, and reported elsewhere. [1-4]

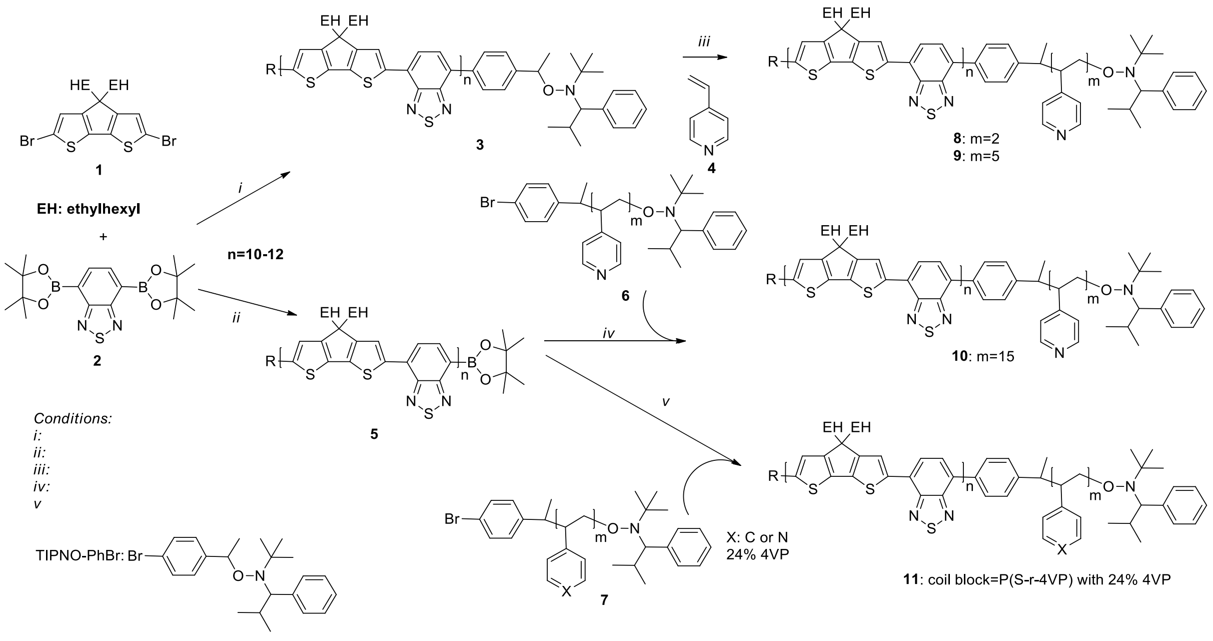

Scheme S1. Synthetic routes for the BCPs used in this work (8 BCP2, 9 BCP5, 10 BCP15, and 11 BCP100, respectively).

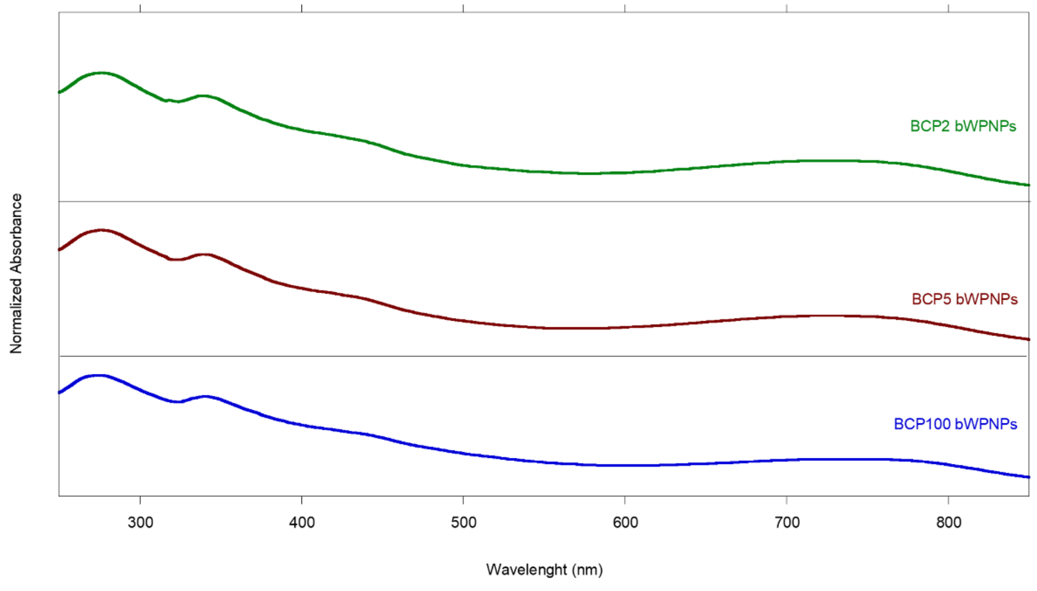

Figure S1. Normalized absorption spectra of BCP2 (green), BCP 5 (red) and BCP100 (blue) bWPNPs dispersion after dilution with water 1:100.

- GIWAXS MEASUREMENTS ON PCBM:BCP=3:1 blend WPNP FILMS

The samples on cleaned glass (1x1 cm^2) were prepared in different ways and properly thermally treated before measurements. Details on measurements are elsewhere reported. The list of samples as well as the main structural features are below specified in **Table S1**.

Table S1. Features of BCP blend WPNP films differently treated

sample [a]	D1[b]	L1	D2[c]	L2	D3[d]	L3
**BCP15 bWPNPs**	0.86	<3	-	-	0.41	<2
**BCP5 bWPNPs**	0.85	3	-	-	0.41	<2
**BCP15 bWPNPs (5 layers)**	0.85	3	-	-	0.41	<2
**BCP15 bWPNPs (annealed at 140 °C)**	1.05 /0.68 [e]	3	-	-	0.41	<2

[a] Both values of d-spacings and L (crystallite dimensions) are expressed in nm.
[b] Attributed to macromolecule aggregation
[c] Attributed to PCBM
[d] Attributed to both glass substrate and disordered copolymer part
[e] the second spacing has been observed in a second measurements after six months, possibly due to copolymer degradation.

All the films are largely amorphous, as clearly indicated by both D3 bump and L1 value in **Table S1** and even in case of repeated annealing the majority of the material remains un-crystallized. The observation of multiple aggregation spacings (BCP15 bWPNP film with 5 layers) is indicative of the materials tendency to poorly ordering, moreover the d-contraction observed in thermally stressed films (BCP15 bWPNP film annealed at 140 °C) indicate that to reach effective molecular aggregation a simple annealing up to PCBM melt is insufficient.

In **Figure S1** XRD profile of BCP15 bWPNP film with 5 layers is shown with the indication of the main features, the poor order achieved yields peaks attributed to copolymer weak and broad, while the amorphous part, glass diffraction and PCBM most intense peak occupy the same spectrum area preventing an efficient deconvolution.

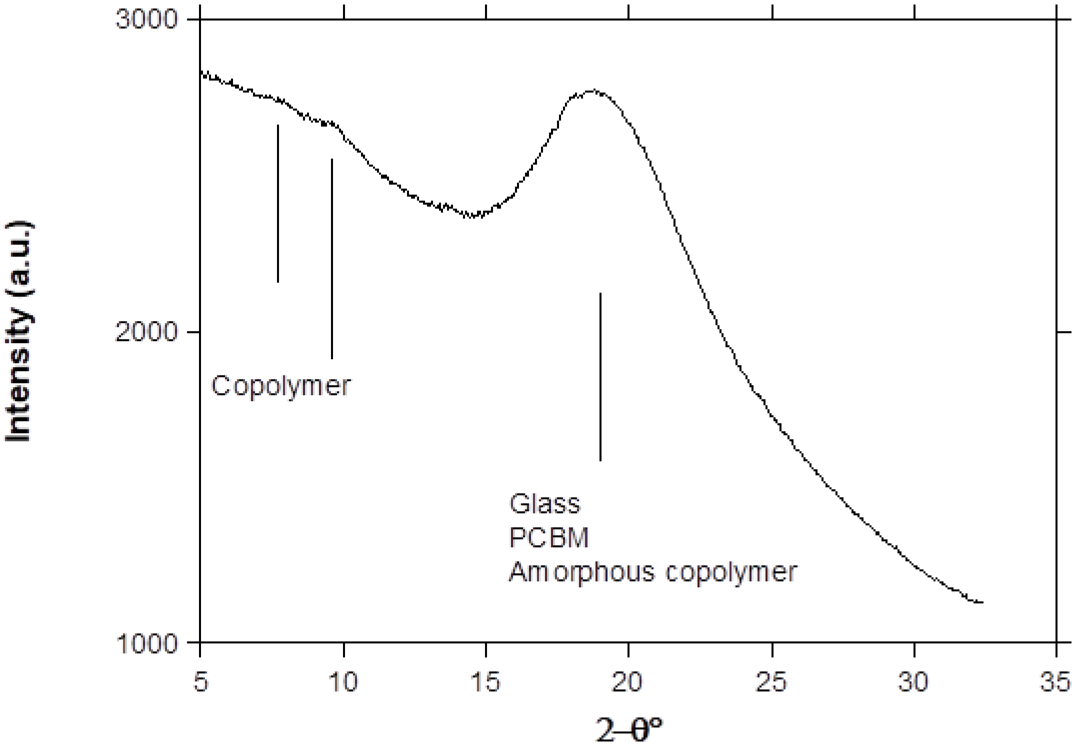

Figure S2. XRD profile extracted from 2D-images of BCP15 bWPNP film with 5 layers, vertical bars indicate diffraction features (see text).

Table S2. Hole mobility calculated through space-charge limited current method of layers deposited from BCP nWPNP suspensions.

WPNP samples (treatment)	hole mobility [$cm^2 \cdot V^{-1} \cdot s^{-1}$]
BCP2 nWPNPs (as casted)	-[a]
BCP2 nWPNPs (annealed 120 °C)	-[a]
BCP5 nWPNPs (as casted)	-[a]
BCP5 nWPNPs (annealed 120 °C)	4.6×10^{-3}
BCP15 nWPNPs (as casted)	1.5×10^{-4}
BCP15 nWPNPs (annealed 90 °C)	3×10^{-3}
BCP100 nWPNPs (annealed 120 °C)	1.3×10^{-4}

[a]resistive

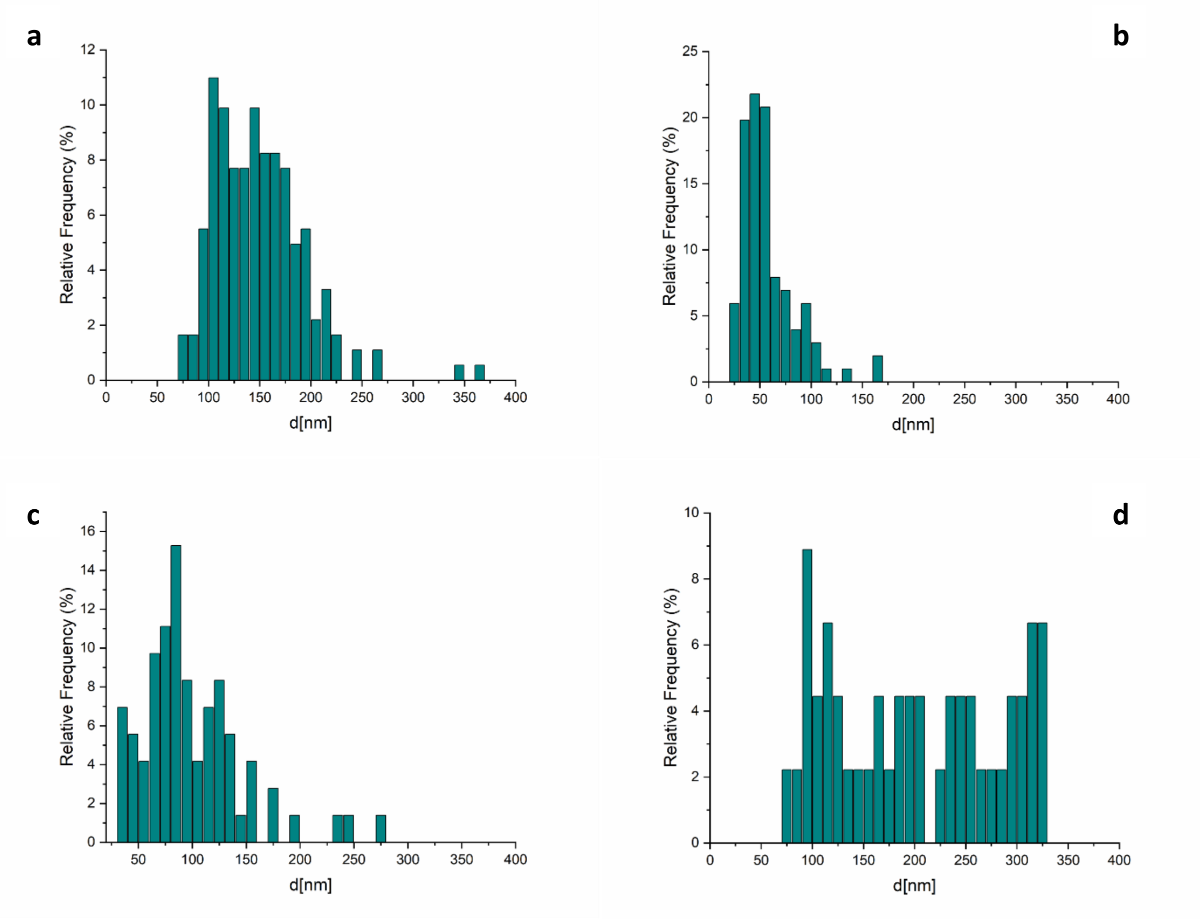

Figure S3. Size distribution of the bWPNPs a) BCP2 bWPNPs b) BCP5 bWPNPs c) BCP15 bWPNPs d) BCP100 bWPNPs.

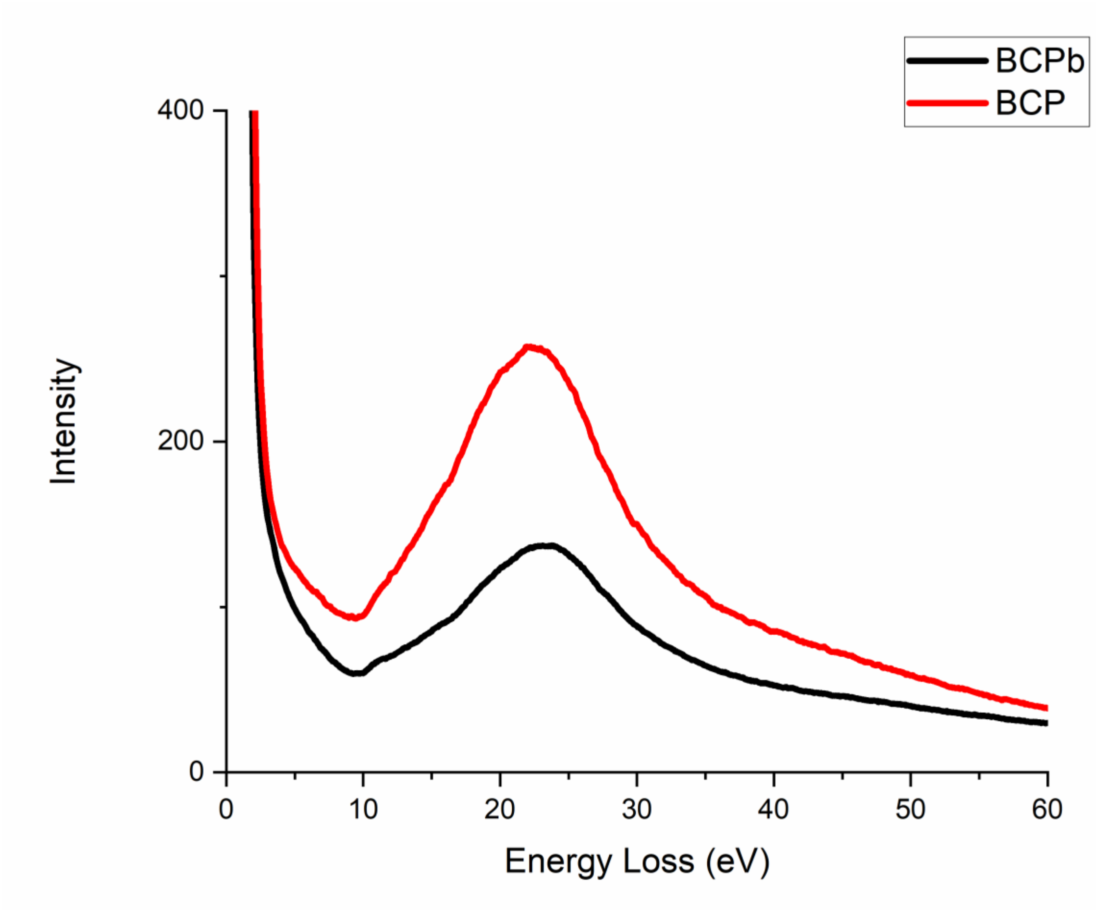

Figure S4. EELS low loss spectra. In red EELS spectra of the nWPNP sample, the plasmon peak is at 22 eV. In black EELS spectra of a BWPNP sample, where the plasmon peak is shifted at 25-26eV because of the superimposition of the nWPNP sample and PCBM plasmon that has the maximum at 30eV [4,5]

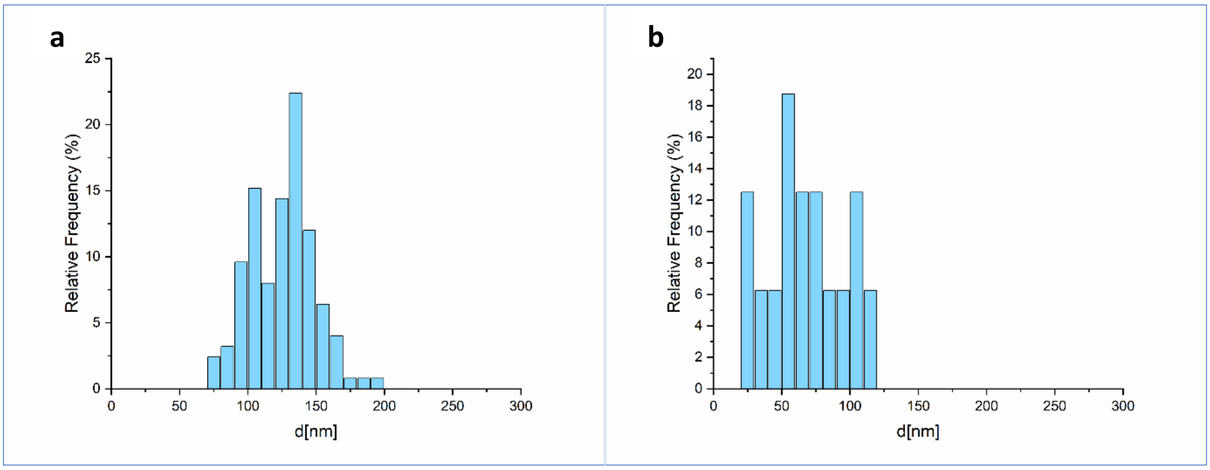

Figure S5. Size distribution of the PCBM rich cores measured for the a) BCP2 bWPNPs b) BCP15 bWPNPs.

References

1. Zappia, S.; Mendichi, R.; Battiato, S.; Scavia, G.; Mastria, R.; Samperi, F.; Destri, S. Characterization of amphiphilic block-copolymers constituted of a low band gap rigid segment (PCPDTBT) and P4VP based coil block synthesized by two different strategies. *Polymer* **2015**, *80*, 245-258, doi:10.1016/j.polymer.2015.10.062.
2. Zappia, S.; Di Mauro, A.E.; Mastria, R.; Rizzo, A.; Curri, M.L.; Striccoli, M.; Destri, S. Rod-coil block copolymer as nanostructuring compatibilizer for efficient CdSe NCs/PCPDTBT hybrid solar cells. *European Polymer Journal* **2016**, *78*, 352-363, doi:10.1016/j.eurpolymj.2016.03.021.
3. Zappia, S.; Scavia, G.; Ferretti, A.M.; Giovanella, U.; Vohra, V.; Destri, S. Water-Processable Amphiphilic Low Band Gap Block Copolymer:Fullerene Blend Nanoparticles as Alternative Sustainable Approach for Organic Solar Cells. *Advanced Sustainable Systems* **2018**, *2*, 10, doi:10.1002/adsu.201700155.
4. Ferretti, A.M.; Zappia, S.; Scavia, G.; Giovanella, U.; Villafiorita-Monteleone, F.; Destri, S. Surfactant-free miniemulsion approach for low band gap rod-coil block copolymer water-processable nanoparticle fabrication: Film preparation and morphological characterization. *POLYMER* **2019**, *174*, 61-69, doi:10.1016/j.polymer.2019.04.055.
5. Dyck, O.; Hu, S.; Das, S.; Keum, J.; Xiao, K.; Khomami, B.; Duscher, G. Quantitative Phase Fraction Detection in Organic Photovoltaic Materials through EELS Imaging. POLYMERS 2015, 7, 2446-2460, doi:10.3390/polym7111523.
